# Which Bacterial Agent Is More Influential in the Development of Retinopathy of Prematurity: Gram-Positive or Gram-Negative?

**DOI:** 10.3390/children13050705

**Published:** 2026-05-20

**Authors:** Batuhan Yeke, Mustafa Senol Akin, Seyma Butun Turk, Sarkhan Elbayiyev, Burak Ceran, Ozdemir Ozdemir, Ufuk Cakir

**Affiliations:** 1 Department of Pediatrics, Division of Neonatology, Ankara Bilkent City Hospital, University of Health Sciences, Ankara 06800, Turkey; 2 Educational-Surgery Clinic, Department of Neonatology, Azerbaijan Medical University, Baku AZ1022, Azerbaijan; 3 Department of Ophthalmology, Ankara Bilkent City Hospital, University of Health Sciences, Ankara 06800, Turkey

**Keywords:** Gram-negative, Gram-positive, neonatal sepsis, premature, retinopathy of prematurity

## Abstract

**Highlights:**

**What are the main findings?**

- Both Gram-positive and Gram-negative neonatal sepsis are independent risk factors for the development of any-stage ROP.
- Gram-positive sepsis is additionally an independent predictor of treatment-requiring ROP, whereas Gram-negative sepsis is not.

**What are the implication of the main findings?**

- The type of neonatal sepsis should be incorporated into ROP risk stratification and screening protocols.
- Targeting the prevention and early management of Gram-positive sepsis may help reduce the progression to severe ROP.

**Abstract:**

**Objective:** In addition to hyperoxia, inflammation and infection contribute to the pathogenesis of retinopathy of prematurity (ROP). However, the differential effects of specific bacterial pathogens on ROP development remain unclear. This study aimed to evaluate the association between sepsis due to Gram-positive and Gram-negative bacteria and the development and severity of ROP. **Materials and Methods:** Infants at risk for ROP, defined as those with birth weight ≤1500 g or gestational age ≤32 weeks, or those with birth weight >1500 g or gestational age >32 weeks who required cardiorespiratory support, were included in this retrospective study. Patients were categorised into no-ROP and any-stage ROP groups, as well as treatment-requiring and non-treatment-requiring ROP groups. The clinical characteristics, laboratory findings, and microbiological data were compared between the groups. **Results:** Among the 319 enrolled infants, 193 (60.6%) did not develop ROP, whereas 126 (39.4%) developed any-stage ROP. Clinical early-onset sepsis, clinical and proven late-onset sepsis, and increased frequency of late-onset sepsis episodes were significantly associated with any-stage and treatment-requiring ROP (*p* < 0.05). In multivariate analysis, both Gram-positive (OR 2.36, 95% CI 1.2–4.6, *p* = 0.012) and Gram-negative bacterial sepsis (OR 3.56, 95% CI 1.5–8.3, *p* = 0.004) were independently associated with any-stage ROP. In addition, Gram-positive bacterial sepsis (OR 4.4, 95% CI 1.1–16.9, *p* = 0.031) was independently associated with treatment-requiring ROP. **Conclusions:** Gram-positive bacterial sepsis was associated with both any-stage ROP and treatment-requiring ROP, whereas Gram-negative bacterial sepsis was associated with any-stage ROP only. These findings support the potential role of pathogen-specific inflammatory processes in ROP development and progression. Further prospective studies incorporating detailed inflammatory and oxygenation parameters are required to clarify these relationships.

## 1. Introduction

Retinopathy of prematurity (ROP) is the leading cause of visual impairment and blindness in preterm infants [1]. The primary pathogenic mechanism underlying ROP development is oxygen-induced retinal injury, in which exposure to supraphysiological oxygen concentrations and high fractions of inspired oxygen delivered to the immature retina lead to excessive reactive oxygen species generation and disruption of normal retinal vascularization. [2,3]. Although prematurity and low birth weight (LBW) are the principal risk factors for this condition, its pathogenesis is multifactorial, with a range of antenatal and postnatal influences contributing to its onset and progression.

Beyond prematurity and LBW, various modifiable and non-modifiable risk factors have been associated with ROP, including fluctuations in oxygen tension (hyperoxia and hypoxia), disturbances in carbon dioxide levels (hypercarbia and hypocarbia), prolonged respiratory support, asphyxia, metabolic acidosis, respiratory distress syndrome (RDS), patent ductus arteriosus (PDA), bronchopulmonary dysplasia (BPD), intraventricular haemorrhage (IVH), necrotizing enterocolitis (NEC), transfusions, erythropoietin administration, and infections [2,4].

Neonatal sepsis is a significant risk factor for ROP [5]. Nevertheless, the precise roles of distinct infectious agents and their pathogenic mechanisms in ROP remain unclear. The outcomes associated with sepsis are heterogeneous and often dictated by the nature of the infective organism, its genetic determinants, and the inflammatory response profile of the host.

Notably, Gram-negative bacterial infections induce a robust systemic inflammatory response, which correlates with an increased risk of multisystem organ dysfunction and mortality. In contrast, Gram-positive infections are generally less severe. Fungal infections tend to occur in infants who are hospitalised for prolonged periods and are extensively exposed to broad-spectrum antibiotics. They are frequently associated with more severe complications than Gram-positive or Gram-negative bacterial infections [6,7,8].

Given the varied clinical manifestations and outcomes of neonatal sepsis according to the infecting pathogen, it is plausible that the risk and course of ROP may also differ according to the type of microorganism involved. However, most previous studies have evaluated sepsis as a single entity, and data on the differential impact of Gram-positive and Gram-negative bacterial infections on ROP severity remain limited [6,7,8]. Furthermore, the potential role of pathogen-specific inflammatory responses in retinal vascular injury has not been fully elucidated. Therefore, this study aimed to identify and assess the risk factors associated with the development of ROP, with a particular focus on delineating the influence of specific microbial pathogens responsible for neonatal sepsis.

## 2. Methods

### 2.1. Study Design

This retrospective study was conducted in a single Neonatal Intensive Care Unit (NICU) and included neonates admitted between January 2023 and March 2024 who met the established criteria for retinopathy of prematurity (ROP) screening. The Bilkent City Hospital Ethics Committee approved this study before its commencement, in accordance with the Declaration of Helsinki.

### 2.2. Study Population

Eligible participants were screened for ROP in accordance with the national guidelines established by the Neonatology and Ophthalmology Society. The inclusion criteria comprised infants with birth weight (BW) ≤ 1500 g, gestational age (GA) ≤ 32 weeks, those identified as high-risk by the medical team, and neonates requiring cardiorespiratory support [1]. The exclusion criteria were death before retinal examination, lack of indication for retinal evaluation, and presence of congenital or chromosomal abnormalities.

### 2.3. ROP Assessment

Retinal examinations were performed and categorised according to the International Classification of Retinopathy of Prematurity (ICROP) [9], segmenting the retina into three zones relative to the optic disc to determine the location and severity of ROP. Management decisions and follow-up schedules adhered to the ICROP and national guidelines [2,9]. Treatment (laser therapy or anti-vascular endothelial growth factor [anti-VEGF] agents) was administered when indicated [1]. Initial comparisons of demographic, clinical, and laboratory parameters were conducted between infants with and without ROP. Subsequent analyses compared the clinical characteristics and laboratory data of treated and untreated ROP cases.

### 2.4. Demographic and Clinical Data Collection

The following variables were recorded for all participants: gestational age, birth weight, sex, Apgar scores at 1 and 5 min, antenatal steroid administration, presence of chorioamnionitis, and delivery via caesarean section. Additionally, major morbidities such as respiratory distress syndrome (RDS), bronchopulmonary dysplasia (BPD), patent ductus arteriosus (PDA), intraventricular haemorrhage (IVH, stage ≥ 3), necrotising enterocolitis (NEC, stage ≥ 2), and mortality were documented. The duration of mechanical ventilation, non-invasive ventilation, supplemental oxygen therapy, and NICU stay were recorded, with respiratory support durations calculated cumulatively from birth until discontinuation of support.

### 2.5. Laboratory-Microbiological Assessment and Definitions of Sepsis

The diagnosis and management of sepsis were consistent with the national protocols. Early-onset sepsis (EOS) was defined as sepsis occurring within the first 72 h of life, whereas late-onset sepsis (LOS) was defined as sepsis occurring after 72 h. Clinical sepsis was defined as the presence of clinical findings compatible with sepsis and abnormal laboratory markers requiring antibiotic treatment despite negative blood cultures. Proven sepsis was defined as the isolation of a pathogenic microorganism from blood cultures obtained during a septic episode [10,11,12,13,14,15]. The Gram-positive, Gram-negative, and fungal sepsis groups were classified according to the blood culture results.

According to our unit protocol, laboratory assessments routinely included complete blood count and blood cultures obtained at NICU admission after birth. In addition, C-reactive protein (CRP), interleukin-6 (IL-6), and procalcitonin (PCT) levels were routinely measured at the 6th hour after birth. In infants with suspected sepsis, complete blood count, blood culture, CRP, IL-6, and PCT were evaluated together with cerebrospinal fluid protein and glucose levels and cerebrospinal fluid culture. These laboratory parameters were routinely obtained as part of standard NICU sepsis evaluation protocols and were retrospectively extracted from medical records. Clinical details regarding the timing and frequency of sepsis and the incidence of meningitis were also collected.

### 2.6. Statistical Analysis

Data analysis was performed using SPSS version 22.0 (SPSS Inc., Chicago, IL, USA). Distribution normality was evaluated using a histogram and the Kolmogorov–Smirnov test. Categorical variables were compared using Fisher’s exact or Pearson’s chi-square tests, and continuous variables were compared using Student’s *t*-test or Mann–Whitney U test, as appropriate. Normally distributed data are reported as mean ± standard deviation, whereas non-normally distributed data are expressed as the median with interquartile range (IQR). Categorical variables were summarised using frequencies and percentages. Univariate and multivariate logistic regression analyses were used to identify the risk factors associated with ROP development and treatment, with results reported as odds ratios (OR) with 95% confidence intervals (CI). The model fit for logistic regression was assessed using the omnibus and Hosmer–Lemeshow goodness-of-fit tests. Statistical significance was set at *p* < 0.05. Variables that demonstrated significance in the initial comparisons or were deemed potential confounders were included in the final regression model.

## 3. Results

### 3.1. Study Population

**Figure 1:**
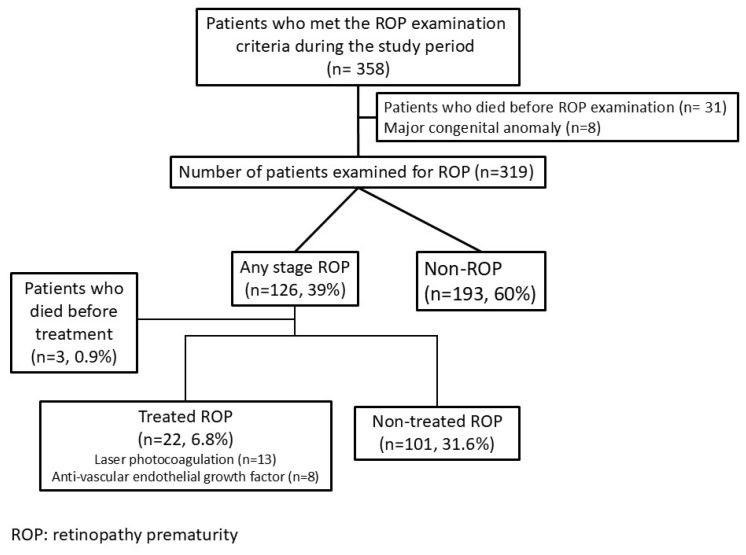
Retinopathy of Prematurity (ROP) screening and management flowchart of our study population.

A total of 319 neonates were enrolled in the study, of whom 117 (36.7%) were male. Retinopathy of prematurity (ROP) was detected in 126 infants (39.4%), of whom 22 required medical or surgical intervention. Specifically, 13 patients underwent laser photocoagulation, and eight received anti-vascular endothelial growth factor (anti-VEGF) therapy (Figure 1).

### 3.2. Comparison Between Patients with and Without ROP

Infants diagnosed with ROP demonstrated significantly higher median interleukin-6 (IL-6) concentrations at birth (43.95 vs. 24.8 pg/mL; *p* = 0.029) and at the onset of sepsis (528 vs. 84.4 pg/mL; *p* = 0.035) than those without ROP. Furthermore, those with ROP had a significantly lower gestational age [median (IQR): 28 (27–29) weeks vs. 30 (29–31) weeks; *p* < 0.001], birth weight [1015 (830–1330) g vs. 1440 (1230–1640) g; *p* < 0.001], and Apgar scores at 1 min (5 vs. 6; *p* < 0.001) and 5 min (7 vs. 8; *p* < 0.001).

These infants also required longer durations of non-invasive ventilation (NIV) [13 vs. 5 days; *p* < 0.001], mechanical ventilation (MV) [12 vs. 0 days; *p* < 0.001], supplemental oxygen therapy (OS) [22 vs. 5 days; *p* < 0.001], and NICU admission [82 vs. 43 days; *p* < 0.001] than those without ROP.

Morbidities, such as respiratory distress syndrome (66.1% vs. 37.5%; *p* < 0.001), bronchopulmonary dysplasia (69% vs. 25%; *p* < 0.001), patent ductus arteriosus (45.2% vs. 22.8%; *p* < 0.001), and severe intraventricular haemorrhage (11.1% vs. 2.6%; *p* < 0.001), were also more prevalent among infants with ROP.

The incidence of clinical early neonatal sepsis (31% vs. 14.5%; *p* < 0.001), clinical sepsis (84.1% vs. 53.4%; *p* < 0.001), and proven late neonatal sepsis (50.8% vs. 24.4%; *p* < 0.001) was significantly higher in the ROP group. Notably, sepsis caused by Gram-positive (32.5% vs. 16.6%; *p* < 0.001) and Gram-negative (17.5% vs. 6.7%; *p* = 0.003) bacteria was significantly more common in infants with ROP than in those without (Table 1). Detailed distributions and frequencies of the isolated microorganisms identified in blood cultures are presented in Supplementary Table S1.

### 3.3. Comparison Between Patients with Treatment-Requiring ROP and the Remaining Cohort

Patients who developed treatment-requiring ROP exhibited substantially elevated IL-6 levels at birth [300 pg/mL vs. 28.1 pg/mL, *p* = 0.004] and, although not statistically significant, higher IL-6 levels at the onset of sepsis [152 pg/mL vs. 41 pg/mL, *p* = 0.44] than infants without treatment-requiring ROP. These infants also presented with significantly lower gestational age [26 (25–28) vs. 30 (28–30) weeks, *p* < 0.001], birth weight [795 (715–940) vs. 1360 (1100–1540) g, *p* < 0.001], and Apgar scores at 1 (4 vs. 6, *p* = 0.001) and 5 min (6 vs. 7, *p* = 0.001).

Infants with treatment-requiring ROP required longer durations of mechanical ventilation [38 (26–46) vs. 1 (0–8) days, *p* < 0.001], non-invasive ventilation [25 (12–36) vs. 6 (2–14) days, *p* < 0.001], supplemental oxygen therapy [33 (27–39) vs. 8 (3–22) days, *p* < 0.001], and NICU hospitalisation [123 (97–133) vs. 52 (38–73) days, *p* < 0.001] than infants without treatment-requiring ROP.

Additional morbidities, including respiratory distress syndrome (86.4% vs. 51%; *p* < 0.001), bronchopulmonary dysplasia (86.4% vs. 37.8%, *p* < 0.001), patent ductus arteriosus (54.5% vs. 29.3%, *p* = 0.013), and severe intraventricular haemorrhage (40.9% vs. 3.1%, *p* < 0.001), as well as higher rates of clinical early neonatal sepsis (50% vs. 19%, *p* = 0.001), clinical late neonatal sepsis (90.9% vs. 63.3%, *p* = 0.009), and confirmed late neonatal sepsis (54.5% vs. 33%, *p* = 0.04), were noted in the treatment-requiring ROP group. In particular, Gram-positive bacterial sepsis was more prevalent in these patients (45.5% vs. 20.7%, *p* = 0.007) (Table 2).

### 3.4. Predictors of ROP Development

Univariate logistic regression analysis identified the following as significant risk factors for ROP: clinical early neonatal sepsis (odds ratio [OR]: 2.642, 95% confidence interval [CI]: 1.523–4.581; *p* = 0.001), clinical late neonatal sepsis (OR: 4.631, 95% CI: 2.658–8.069; *p* < 0.001), proven late neonatal sepsis (OR: 3.207, 95% CI: 1.985–5.180; *p* < 0.001), Gram-positive bacterial sepsis (OR: 2.427, 95% CI: 1.426–4.130; *p* = 0.001), Gram-negative bacterial sepsis (OR: 2.929, 95% CI: 1.416–6.059; *p* = 0.004), increased number of late neonatal sepsis episodes (OR: 2.615, 95% CI: 1.767–3.872; *p* < 0.001), and foetal inflammatory response syndrome (FIRS) (OR: 3.008, 95% CI: 1.165–7.763; *p* = 0.023) (Table 3).

In the multivariate analysis, gestational age (OR: 0.623, 95% CI: 0.509–0.763; *p* < 0.001), bronchopulmonary dysplasia (OR: 2.492, 95% CI: 1.325–4.689; *p* = 0.005), Gram-positive bacterial sepsis (OR: 2.361, 95% CI: 1.207–4.620; *p* = 0.012), and Gram-negative bacterial sepsis (OR: 3.568, 95% CI: 1.518–8.386; *p* = 0.004) emerged as independent predictors of ROP development.

### 3.5. Predictors of Treatment-Requiring ROP

Univariate analysis indicated that clinical early neonatal sepsis (OR: 4.25, 95% CI: 1.754–10.279; *p* = 0.001), clinical late neonatal sepsis (OR: 5.806, 95% CI: 1.331–25.323; *p* = 0.019), proven late neonatal sepsis (OR: 2.437, 95% CI: 1.017–5.839; *p* = 0.046), Gram-positive bacterial sepsis (OR: 3.183, 95% CI: 1.313–7.715; *p* = 0.01), and FIRS (OR: 3.819, 95% CI: 1.156–12.617; *p* = 0.028) were significantly associated with treatment-requiring ROP, whereas Gram-negative bacterial sepsis was not (OR: 0.791, 95% CI: 0.177–3.537; *p* = 0.759) (Table 4).

Multivariate logistic regression analysis identified lower gestational age (OR: 0.507, 95% CI: 0.357–0.718; *p* < 0.001), severe intraventricular haemorrhage (OR: 16.469, 95% CI: 3.402–79.733; *p* < 0.001), and Gram-positive bacterial sepsis (OR: 4.407, 95% CI: 1.146–16.949; *p* = 0.031) as independent risk factors for developing treatment-requiring ROP (Table 4).

## 4. Discussion

In this cohort of preterm infants born before 32 weeks, lower gestational age, birth weight, Apgar scores, and insufficient weight gain during the first 28 days were significant risk factors for ROP development and treatment-requiring ROP. Elevated IL-6 levels at birth, increased early neonatal sepsis, clinically and proven late neonatal sepsis, and more late sepsis episodes were significant factors for any-stage ROP and treatment-requiring ROP. Although Gram-positive and Gram-negative sepsis were more frequent in infants with any stage of ROP, only Gram-positive bacterial sepsis was associated with treated ROP. Gram-negative sepsis did not increase the risk of treated ROP, and fungal infections were not significantly associated with any ROP stage.

Multivariate analysis showed that lower gestational age, BPD, and Gram-positive or Gram-negative bacterial sepsis were independent predictors of ROP. Factors independently associated with treatment-requiring ROP included lower gestational age, Gram-positive bacterial sepsis, and severe intraventricular haemorrhage (IVH).

Low gestational age, low birth weight, and hyperoxia remain the primary risk factors for ROP [16]. Retinopathy of prematurity develops through two phases: an initial hyperoxic injury to the immature retina with cessation of vascularization, followed by a hypoxic phase in which increased metabolic demand leads to the upregulation of angiogenic factors and aberrant neovascularization. This cascade may ultimately result in retinal detachment and vision loss [16]. Although these risk factors are well established, the exact molecular and environmental mechanisms underlying ROP development remain unclear. Prenatal and postnatal infections have also been implicated in ROP pathogenesis, potentially influencing disease onset and severity through inflammatory mediators.

Preterm neonates with low birth weight face an increased risk of ROP and other morbidities, including low Apgar scores, RDS, BPD, PDA, IVH, NEC, poor weight gain, and prolonged intensive care. These findings were confirmed in our cohort, in which complications were elevated among infants with ROP [16,17,18,19]. Our data showed that BPD is an independent risk factor for ROP, reflecting the effect of oxygen exposure. Severe IVH, but not BPD, emerged as an independent risk factor for treatment-requiring ROP, suggesting that severe postnatal complications affect disease progression.

In addition to inflammatory injury, sepsis and severe pulmonary disease may contribute to substantial fluctuations in oxygenation and arterial oxygen tension. Pneumonia, respiratory insufficiency, pulmonary hypertension, prolonged respiratory support, and impaired gas exchange during septic episodes may expose the immature retina to repeated hyperoxic and hypoxic insults. These oxygen fluctuations may further amplify dysregulated retinal angiogenesis, contributing to ROP progression.

Infectious and inflammatory processes likely interrupt retinal vascularization during ROP’s hyperoxic first phase of ROP and promote pathological neovascularization in the hypoxic phase. However, the impact of sepsis on the transition between stages remains inadequately defined [16,17]. Understanding the relationship between infection and ROP pathophysiology is essential, as it may inform risk stratification and enable targeted preventive strategies. Delineating the molecular pathways linking sepsis to aberrant angiogenesis may yield new therapeutic options [16,20,21].

Our findings indicate that early neonatal sepsis, clinical late neonatal sepsis, and proven late neonatal sepsis are significantly associated with treated ROP development. These results align with previous reports linking sepsis to an increased risk of ROP [16,17,18,19]. Postnatal sepsis promotes systemic inflammation through cytokines and growth factors, which disrupt retinal angiogenesis and vascular development [16]. Chorioamnionitis was not associated with ROP, supporting the notion that prenatal inflammation may have a limited effect due to temporal dissociation from retinal vascular development [16,17]. Our results showed a positive association between late neonatal sepsis episodes and ROP development, supporting the notion that repeated inflammatory insults increase the risk of ROP [20]. Infants with even one late-onset neonatal sepsis episode may require heightened ROP surveillance, although prospective studies are required to validate this approach [16,20,21].

Beyond the general influence of infection, previous studies have explored the contribution of specific bacterial and fungal pathogens to the risk of ROP. For example, Cantey et al. described an increased risk of ROP in very low-birth-weight infants with coagulase-negative staphylococcal sepsis [22], while Karlowicz and Noyola et al. attributed adverse retinal outcomes to fungal infections [23,24]. Proinflammatory proteins and angiogenic factors have been implicated as mediators of the infection-related risk of ROP [25,26]. However, few studies have investigated the distinct effects of Gram-positive, Gram-negative, and fungal sepsis on the risk and severity of ROP, and an important gap was highlighted in a recent meta-analysis [25].

The data revealed no relationship between fungal infections or meningitis and ROP, likely due to small sample sizes. Gram-positive bacterial sepsis was identified as a significant risk factor for both any-stage and treated ROP, whereas Gram-negative bacterial sepsis was associated with any-stage ROP. Higher IL-6 levels observed in infants with ROP may reflect increased systemic inflammatory activity during neonatal sepsis. However, because IL-6 was measured at limited time points and longitudinal cytokine dynamics were not evaluated, these findings should be interpreted cautiously. In addition, serum inflammatory biomarkers may not directly represent local retinal inflammatory processes [16,20,26,27].

Although Gram-negative bacterial sepsis is frequently associated with more fulminant clinical presentations, including septic shock and high mortality due to robust inflammatory responses and endotoxemia [27], Gram-positive infections may result in prolonged subclinical inflammation. Variations in bacterial genetics and pathogenicity, rapid clinical onset, and the clinician’s ability to promptly diagnose and treat infections likely modulate the magnitude and duration of systemic inflammation [10]. One possible explanation is that prolonged inflammatory exposure during clinically less fulminant Gram-positive infections may contribute differently to retinal vascular injury and increase the risk of ROP, although this hypothesis requires mechanistic validation. Bhargava et al. reported higher mortality among neonates with Gram-positive sepsis than among those with Gram-negative sepsis [28].

To our knowledge, this study is the first to evaluate the differential contributions of Gram-positive and Gram-negative bacterial sepsis to ROP onset and progression. Associations between late neonatal sepsis frequency, Gram positivity, ROP of any stage or requiring treatment, Gram negativity, and any-stage ROP are supported by pathophysiological and epidemiological observations. The frequency and persistence of septic episodes appear to be crucial for the progression to treatment-requiring ROP, although the precise mechanisms remain unclear and require further investigation, including detailed cytokine profiling.

The limitations of this study include its retrospective, single-centre design, lack of detailed longitudinal data on cytokine levels, and the inability to link sepsis severity to ROP stage and zone. However, the strengths of this study were its large sample size and the novel evaluation of the effects of Gram-positive and Gram-negative bacterial sepsis on ROP. We were unable to evaluate longitudinal oxygenation parameters, such as PaO2 levels or oxygen saturation fluctuations, which may interact with inflammatory pathways during ROP development. Lastly, our study has some statistical limitations that warrant consideration. Although our total cohort size is substantial (n = 319), the relatively small number of infants who required ROP treatment (n = 22) limits the statistical power and robustness of our multivariate logistic regression conclusions for this specific subgroup. Due to this small sample size, the regression models for treatment-requiring ROP should be interpreted with caution regarding residual confounding from baseline severity factors such as extreme prematurity and prolonged oxygen exposure. Future large-scale, multicenter prospective studies are necessary to confirm the independent predictive value of pathogen-specific sepsis in severe, treatment-requiring ROP cases.

Infants with severe prematurity and prolonged respiratory support are inherently at increased risk for both neonatal sepsis and ROP. Therefore, despite multivariate adjustment, residual confounding related to cumulative oxygen exposure, respiratory instability, and overall illness severity cannot be completely excluded.

In conclusion, our findings demonstrate the impact of repeated late neonatal sepsis episodes, Gram-positive bacterial sepsis, and Gram-negative bacterial sepsis on ROP development. These findings suggest that sepsis type and recurrent septic episodes may contribute to ROP risk stratification and warrant further investigation in larger prospective studies. Personalised screening and preventive interventions based on infectious exposure may help reduce ROP-related morbidities. Neonatal sepsis prevention should be prioritised to mitigate the risk of ROP and improve visual outcomes in preterm infants.

## Figures and Tables

**Table 1:** Demographic and clinical characteristics, laboratory findings, and blood culture results of patients with and without retinopathy of prematurity.

Characteristics	Non-ROP (n = 193)	Any-Stage ROP (n = 126)	*p*-Value
Gestational age, weeks	30 (29–31)	28 (27–29)	**<0.001**
Birth weight, g	1440 (1230–1640)	1015 (830–1330)	**<0.001**
SGA, n (%)	18 (9.3)	20 (15.9)	0.078
Male gender, n (%)	117 (60.6)	62 (49.2)	**0.045**
Apgar score 1st min	6 (5–6)	5 (4–6)	**<0.001**
Apgar score 5th min	8 (7–8)	7 (6–8)	**<0.001**
Antenatal steroid administration, n (%)	141 (74.2)	88 (72.7)	0.772
Multiple gestation, n (%)	47 (24.7)	39 (32)	0.163
PPROM, n (%)	40 (20.7)	22 (17.5)	0.471
Chorioamnionitis, n (%)	13 (6.7)	7 (5.6)	0.671
Caesarean section, n (%)	161 (83.4)	112 (88.9)	0.174
hdPDA, n (%)	44 (22.8)	57 (45.2)	**<0.001**
Inotrope use in the first week, n (%)	17 (9)	25 (19.8)	**0.006**
RDS, n (%)	82 (44.1)	84 (67.7)	**<0.001**
BPD, n (%)	48 (25)	87 (69)	**<0.001**
Duration of MV, days	0 (0–3)	12 (1–34)	**<0.001**
Duration of NIV, days	5 (2–10)	13 (6–25)	**<0.001**
Duration of O_2_, days	5 (2–16)	22 (8–33)	**<0.001**
IVH (stage ≥3), n (%)	5 (2.6)	14 (11.1)	**0.002**
NEC (stage ≥2), n (%)	6(3.1)	7 (5.6)	0.28
First 28 days weight gain, g/day	13.93 (9.64–17.68)	10.45 (6.52–13.13)	**<0.001**
Weight gain until discharge, g/day	18.5 (14.6–22.5)	17.2 (13.9–20.9)	0.062
NICU stay, days	43 (34–57)	82 (60–112)	**<0.001**
Mortality, n (%)	12 (6.2)	10 (7.9)	0.554
**Laboratory and culture results**			
C-reactive protein levels at birth (mg/L)	0.8 (0.5–3.1)	1.5 (0.5–3.1)	0.524
Interleukin-6 levels at birth (pg/mL)	24.8 (12–71.2)	43.95 (14.4–177.1)	**0.029**
Procalcitonin levels at birth (ng/mL)	1.13 (0.65–3.68)	1.7(0.53–5.76)	0.435
Clinical early neonatal sepsis, n (%)	28 (14.5)	39 (31)	**<0.001**
Proven early neonatal sepsis, n (%)	0 (0)	0 (0)	-
FIRS, n (%)	7 (3.7)	13 (10.3)	**0.018**
C-reactive protein levels at the onset of sepsis (mg/L)	21 (3–43)	32 (11–75)	0.152
Interleukin-6 levels at the onset of sepsis (pg/mL)	84.4 (13.2–1500)	528 (31.2–1500)	**0.035**
Procalcitonin levels at the onset of sepsis (ng/mL)	0.77 (0.32–4.73)	1.86 (0.42–11.96)	0.091
Clinical late neonatal sepsis, n (%)	103 (53.4)	106 (84.1)	**<0.001**
Proven late neonatal sepsis, n (%)	47 (24.4)	64 (50.8)	**<0.001**
Blood culture results			
Gram-positive bacteria, n (%)	32 (16.6)	41(32.5)	**0.001**
Gram-negative bacteria, n (%)	13 (6.7)	22 (17.5)	**0.003**
Fungal infection, n (%)	2 (1)	1 (0.8)	0.826
The number of late neonatal sepsis	1 (0–1)	1 (1–1)	**<0.001**
Day of first onset of late neonatal sepsis	10 (7–15)	11 (7–15)	0.792
Meningitis, n (%)	6 (3.1)	7 (5.6)	0.280
Protein levels in cerebrospinal fluid	104 (91.6–129.8)	123.5 (98.1–164.9)	0.054
Glucose levels in cerebrospinal fluid	63.5 (51–78)	74.5 (50.5–96)	0.156

Data shown as n (%) or median (IQR). BPD: bronchopulmonary dysplasia; FIRS: Foetal Inflammatory Response Syndrome; IVH: intraventricular haemorrhage; MV: mechanical ventilation; NEC: necrotising enterocolitis; NICU: neonatal intensive care unit; NIV: noninvasive ventilation; hdPDA: hemodynamic patent ductus arteriosus; PPROM: Preterm Premature Rupture of Membranes; RDS: respiratory distress syndrome; ROP: retinopathy of prematurity; SGA: Small for gestational age.

**Table 2:** Demographic and clinical characteristics, laboratory findings, and blood culture results in patients with treatment-requiring retinopathy of prematurity and the rest of the cohort.

Characteristics	Non-Treated ROP and Non-ROP (n = 294)	Treated ROP (n = 22)	*p*-Value
Gestational age, weeks	30 (28–30)	26 (25–28)	**<0.001**
Birth weight, g	1360 (1100–1540)	795 (715–940)	**<0.001**
SGA, n (%)	33 (11.2)	4 (18.2)	0.328
Male gender, n (%)	168 (57.1)	9 (40.9)	0.139
Apgar score 1st min	6 (4–6)	4 (3–4)	**0.001**
Apgar score 5th min	7 (6–8)	6 (5–6)	**0.001**
Maternal age, year	29 (24–35)	27 (25–32)	0.893
Antenatal steroid administration, n (%)	211 (73.5)	15 (71.4)	0.834
Multiple gestation, n (%)	80 (27.8)	6 (28.6)	0.938
PPROM, n (%)	57 (19.4)	3 (13.6)	0.507
Chorioamnionitis, n (%)	19 (6.5)	1 (4.5)	0.722
Caesarean section, n (%)	252 (85.7)	19 (86.4)	0.933
PDA, n (%)	86 (29.3)	12 (54.5)	**0.013**
Inotrope use in the first week, n (%)	32 (11)	8 (36.4)	**0.001**
RDS, n (%)	150 (51)	19 (86.4)	**<0.001**
BPD, n (%)	111 (37.8)	19 (86.4)	**<0.001**
Duration of MV, days	1 (0–8)	38 (26–46)	**<0.001**
Duration of NIV, days	6 (2–14)	25 (12–36)	**<0.001**
Duration of O_2_, days	8 (3–22)	33 (27–39)	**<0.001**
IVH (stage ≥3), n (%)	9 (3.1)	9 (40.9)	**<0.001**
NEC (stage ≥2), n (%)	12 (4.1)	1 (4.5)	0.91
First 28 days weight gain, g/day	12.5 (9.11–16.4)	4.5 (2.8–8.9)	**<0.001**
Weight gain until discharge, g/day	18.2 (14.5–21.5)	17.6 (11.8–19.6)	0.102
NICU stay, days	52 (38–73)	123 (97–133)	**<0.001**
Mortality, n (%)	19 (6.5)	0 (0)	0.219
**Laboratory and culture results**			
C-reactive protein levels at birth (mg/L)	0.8 (0.5–3.1)	3.1 (0.5–3.1)	0.138
Interleukin-6 levels at birth (pg/mL)	28.1 (13.1–82.1)	300 (53–519)	**0.004**
Procalcitonin levels at birth (ng/mL)	1.58 (0.68–3.8)	1.31 (0.73–7.9)	0.993
Clinical early neonatal sepsis, n (%)	56 (19)	11 (50)	**0.001**
Proven early neonatal sepsis, n (%)	0 (0)	0 (0)	-
FIRS, n (%)	16 (5.5)	4 (18.2)	**0.019**
C-reactive protein levels at the onset of sepsis (mg/L)	25 (11–60)	41 (11–75)	0.553
Interleukin-6 levels at the onset of sepsis (pg/mL)	152 (27–1500)	41 (25–1000)	0.44
Procalcitonin levels at the onset of sepsis (ng/mL)	0.99 (0.23–6.49)	3.28 (0.4–22.45)	0.38
Clinical late neonatal sepsis, n (%)	186 (63.3)	20 (90.9)	**0.009**
Proven late neonatal sepsis, n (%)	97 (33)	12 (54.5)	**0.04**
Blood culture results			
Gram-positive bacteria, n (%)	61 (20.7)	10 (45.5)	**0.007**
Gram-negative bacteria, n (%)	33 (11.2)	2 (9.1)	0.758
Fungal infection, n (%)	3 (1)	0 (0)	0.634
The number of late neonatal sepsis	1 (0–1)	1 (1–1)	**0.014**
Day of first onset of late neonatal sepsis	11 (8–8)	8 (5–14)	0.201
Meningitis, n (%)	13 (4.4)	0 (0)	0.31
Protein levels in cerebrospinal fluid	109 (92.9–137.8)	124.2 (65–130.9)	0.96
Glucose levels in cerebrospinal fluid	65 (51–84)	73 (39–120)	0.757

Data are shown as n (%) or median (IQR). BPD, bronchopulmonary dysplasia; FIRS: foetal inflammatory response syndrome; IVH, intraventricular haemorrhage; MV, mechanical ventilation; NEC, necrotising enterocolitis; NICU, neonatal intensive care unit; NIV, noninvasive ventilation; PDA, patent ductus arteriosus; PPROM: Preterm Premature Rupture of Membranes; RDS, respiratory distress syndrome; ROP, retinopathy of prematurity; SGA: Small for gestational age.

**Table 3:** Univariate and multivariate logistic regression analyses for possible predictors of any-stage ROP.

Variables	Univariate				Multivariate			
	aOR	95% Cl		*p*	aOR	95% Cl		*p*
		Lower	Upper			Lower	Upper	
Gestational age	0.527	0.445	0.623	**<0.001**	0.623	0.509	0.763	**<0.001**
BPD	6.26	3.81	10.27	**<0.001**	2.492	1.325	4.689	**0.005**
Gram (+) bacteria	2.427	1.426	4.130	**0.001**	2.361	1.207	4.62	**0.012**
Gram (−) bacteria	2.929	1.416	6.059	**0.004**	3.568	1.518	8.386	**0.004**
Male gender	0.629	0.400	0.990	**0.045**	0.598	0.338	1.058	0.077
RDS	2.57	1.61	4.21	**<0.001**	1.053	0.538	1.987	0.874
hdPDA	2.79	1.72	4.54	**<0.001**	0.992	0.469	2.097	0.984
Severe IVH	4.7	1.64	13.39	**0.004**	1.016	0.237	4.347	0.983
First 28 days weight gain	0.96	0.92	1.004	0.08	0.971	0.927	1.017	0.208
Inotrope use in the first week	2.50	1.29	4.86	**0.007**	0.697	0.276	1.764	0.446
Apgar 5th min	0.61	0.50	0.75	**<0.001**	0.816	0.632	1.055	0.12
FIRS	3.008	1.165	7.763	**0.023**	1.977	0.618	6.331	0.251
Clinical early neonatal sepsis	2.642	1.523	4.581	**0.001**				
Clinical late neonatal sepsis	4.631	2.658	8.069	**<0.001**				
Proven late neonatal sepsis	3.207	1.985	5.180	**<0.001**				

BPD, bronchopulmonary dysplasia; FIRS: foetal inflammatory response syndrome; IVH, intraventricular haemorrhage; Cl, confidence interval; hdPDA, hemodynamic patent ductus arteriosus; aOR, Adjusted Odds ratio; PH, pulmonary hypertension; RDS: Respiratory distress syndrome; ROP, retinopathy of prematurity; Model fitting tests for any-stage ROP: Omnibus test—chi-square = 97.148, *p* < 0.001; Hosmer–Lemeshow test—chi-square = 11.844, *p* = 0.158; Nagelkerke R square = 0.391.

**Table 4:** Multivariate logistic regression analyses for possible predictors of ROP treatment.

Variables	Univariate				Multivariate			
	OR	95% Cl		*p*	aOR	95% Cl		*p*
		Lower	Upper			Lower	Upper	
Gestational age	0.484	0.375	0.624	**<0.001**	0.507	0.357	0.718	**<0.001**
BPD	10.44	3.02	36.09	**<0.001**	2.69	0.27	26.818	0.399
Gram (+) bacteria	3.183	1.313	7.715	**0.01**	4.407	1.146	16.949	**0.031**
Gram (−) bacteria	0.791	0.177	3.537	0.759	0.705	0.065	7.601	0.773
Male gender	0.519	0.215	1.253	0.145	0.558	0.137	2.273	0.415
RDS	6.08	1.76	20.98	**0.004**	3.526	0.371	35.54	0.273
hdPDA	2.90	1.20	6.96	**0.017**	0.421	0.093	1.91	0.262
Severe IVH	21.92	7.45	64.44	**<0.001**	16.469	3.402	79.73	**<0.001**
First 28 days weight gain	0.83	0.76	0.91	**<0.001**	0.932	0.826	1.05	0.247
Inotrope use in the first week	4.6	1.79	11.83	**0.002**	1.459	0.313	6.809	0.631
Apgar 5th min	0.46	0.33	0.65	**<0.001**	0.626	0.37	1.057	0.08
FIRS	3.819	1.156	12.617	**0.028**	1.819	0.355	9.332	0.473
Clinical early neonatal sepsis	4.25	1.754	10.279	**0.001**				
Clinical late neonatal sepsis	5.806	1.331	25.323	**0.019**				
Proven late neonatal sepsis	2.437	1.017	5.839	**0.046**				

BPD, bronchopulmonary dysplasia; FIRS: foetal inflammatory response syndrome; IVH, intraventricular haemorrhage; Cl, confidence interval; hdPDA, hemodynamic patent ductus arteriosus; aOR, Adjusted Odds ratio; PH, pulmonary hypertension; RDS: Respiratory distress syndrome; ROP, retinopathy of prematurity. Model fitting tests for the treatment of ROP: Omnibus test—chi-square = 61.902, *p* <0.001; Hosmer–Lemeshow test—chi-square = 6.891, *p* = 0.541; Nagelkerke R-square = 0.539.

## Data Availability

The original contributions presented in this study are included in the article/Supplementary Materials. Further inquiries should be directed to the corresponding authors.

## Supplementary Materials

The following supporting information can be downloaded at: https://www.mdpi.com/article/10.3390/children13050705/s1, Table S1: Microorganisms identified in positive cultures.
