# Supplementary material for: Which Bacterial Agent Is More Influential in the Development of Retinopathy of Prematurity: Gram-Positive or Gram-Negative?
Source: Children (Basel). 2026 May 20;13(5):705. doi: 10.3390/children13050705 (PMC13204034; doi:10.3390/children13050705)
Supplement: Supplementary file 1 [file children-13-00705-s001.zip › children-4323065-supplementary.pdf]

Microorganisms identified in positive cultures	
**Gram positive bacteria**	**n**
Staphylococcus epidermidis	55
Staphylococcus haemolyticus	2
Staphylococcus capitis	8
Staphylococcus aureus	1
Staphylococcus hominis	4
Micrococus luteus	1
Enterococcus faecalis	2
**Gram negative bacteria**	
Serratia marcescens	7
Serratia liquefaciens	1
Raoultella ornithinolytica	1
Pseudomonas aeruginosa	1
Klebsiella pneumoniae	12
Klebsiella oxytoca	5
Enterobacter cloacae	5
Escherichia coli	1
Acinetobacter lwoffii	1
Acinetobacter baumannii	1
**Fungal infection**	
Candida glabrata	3
